# Impact of adjuvant therapy on outcomes of cancer of the stomach and gastroesophageal junction in the real-world

**DOI:** 10.1007/s10120-025-01624-8

**Published:** 2025-05-16

**Authors:** Steffen M. Heckl, Hans-Michael Behrens, Ulrike Ebert, Dita Ulase, Florian Richter, Thomas Becker, Anne Letsch, Christoph Röcken

**Affiliations:** 1 Department of Pathology, Christian-Albrechts-University, University Hospital Schleswig-Holstein, Arnold-Heller-Str. 3, Building U33, 24105 Kiel, Germany; 2 Department of Internal Medicine II, Christian-Albrechts-University, University Hospital Schleswig-Holstein, Kiel, Germany; 3 Department of General, Visceral, Thoracic, Transplant and Pediatric Surgery, Christian-Albrechts-University, University Hospital Schleswig-Holstein, Kiel, Germany

**Keywords:** Gastric cancer, Esophagogastric junction, Chemotherapy, Adjuvant, Biomarkers

## Abstract

**Background:**

Since the FLOT4 gastric cancer (GC) trial, the use of adjuvant chemotherapy has been perceived as limited and its added value questioned. We wanted to objectify this perception and reassess the value of adjuvant chemotherapy in a real-world setting.

**Methods:**

In our retrospective cohort study we analyzed real-world data from 147 patients with GC or cancer of the gastroesophageal junction (AEG) who received perioperative FLOT. Data originated from clinical care at the university hospital, local hospitals and medical practices. Clinicopathologic data, survival outcomes, and targetable biomarkers were analyzed.

**Results:**

Median overall survival (OS) and tumor specific survival (TSS) were 19.4 ± 2.9 and 19.9 ± 3.1 months, respectively. 84.4% completed all cycles of neoadjuvant chemotherapy. The pathological complete response rate was 11.8%. Adjuvant chemotherapy was initiated in only 42.9%. Survival rates of patients with marked tumor regression (TRG1) were not improved by adjuvant chemotherapy. Conversely, patients with partial histopathologic response (TRG2) showed a marked trend and those with minimal histopathologic response (TRG3) showed a significantly longer survival with any number of adjuvant chemotherapy cycles (OS: 22.3 ± 2.6 months versus 8.7 ± 2.4 months, *p* = 0.005; TSS: 22.3 ± 4.5 months versus 8.7 ± 2.4 months, *p* = 0.016). Targetable biomarkers PD-L1, Claudin 18.2, HER2 and microsatellite instability were detected in 53.4%, 26.2%, 7.8%, and 3.9% of the TRG2/3 patient subset, respectively.

**Conclusions:**

In the real-world setting, adjuvant chemotherapy proved to be a critical turning point of the FLOT regimen. It should be sought—even in a reduced form—in patients with TRG2/3.

**Supplementary Information:**

The online version contains supplementary material available at 10.1007/s10120-025-01624-8.

## Introduction

Worldwide, gastric cancer (GC) is the fifth most common human malignancy with a 5–10 year survival rate of less than 40% [1]. Until 18 years ago, surgical resection was the only curative treatment option. The MAGIC trial published in 2006 introduced the perioperative chemotherapy regimen with ECF (epirubicin, cisplatin, fluorouracil) [2] and represented a first breakthrough in oncological treatment. In 2019, this protocol was challenged in the FLOT4 trial [3] with the perioperative FLOT regimen for locally advanced, resectable GC or adenocarcinomas of the esophagogastric junction (AEG). The FLOT protocol consists of four neoadjuvant and four adjuvant chemotherapy cycles of fluorouracil, leucovorin, oxaliplatin and docetaxel. The five-year survival rate improved from 36% with perioperative ECF to 48% with perioperative FLOT.

In spite of all the progress that has been made with the perioperative FLOT regimen, the use of the adjuvant phase is perceived to be limited in the real-world setting. Adjuvant chemotherapy is often less tolerated and therefore many oncologists are inclined to omit the adjuvant phase. Therefore, we wanted to objectify and evaluate the impact of adjuvant chemotherapy on survival. We hypothesized that omitting the adjuvant phase would eliminate the survival benefit. A rigorous consideration of the issue leads to another critical question: Should all patients receive adjuvant chemotherapy whenever possible, and if not, which subgroup should be targeted? As we hypothesized that patients with only partial, minimal or no tumor regression after neoadjuvant chemotherapy with FLOT may especially benefit from targeted therapies in the future, we wanted to know what the biomarker profile of this critical subset of patients would be.

We tested the following central hypotheses: (1) omission of adjuvant chemotherapy is frequent in the real-world setting; (2) the survival benefit of the FLOT regimen is significantly reduced when the use of adjuvant chemotherapy is low; (3) the impact of adjuvant chemotherapy on survival is associated with the histopathological tumor regression grade. In addition, (4) we wanted to map the landscape of targetable biomarkers in the critical subset of patients with only partial, minimal or no tumor regression.

## Methods

### Study design

In our retrospective cohort study, we analyzed a contemporary group of patients who received the perioperative chemotherapy regimen FLOT. All patients were from the same geographic area and had similar ethnic and socioeconomic backgrounds. The surgery was performed at the University Hospital of Schleswig–Holstein, Kiel, Germany. Perioperative chemotherapy was given in various medical institutions, such as various private practices and regional hospitals, which all belong to the recruitment area of our University Hospital. The study did not receive any third-party funding, e.g. by pharmaceutical companies. The study was conducted by a multidisciplinary team of pathologists, oncologists, surgeons and a statistician.

### Patients

All patients who underwent total or partial gastrectomy or esophagectomy for adenocarcinoma of the stomach or esophago-gastric junction between 2010 and 2022 were retrieved from the archive of the Department of Pathology, University Hospital Schleswig–Holstein, Kiel, Germany. Resection specimens were obtained during routine therapeutic procedures. Patients, who were treated with perioperative chemotherapy regimens, including historical regimens, were identified.

Patients were included if (1) adenocarcinoma of the stomach or esophagogastric junction was histologically confirmed and (2) at least one chemotherapy cycle of the FLOT regimen was performed with a curative intent. Exclusion criteria were defined as (1) histologically identified tumor type other than adenocarcinoma, (2) metastatic or unresectable GC/AEG at the time of diagnosis, (3) T1 N0 stage tumors, as perioperative chemotherapy is not required, (4) patients not eligible for surgery, and (5) a recent synchronous cancer diagnosis of another primary. Each resected specimen was grossly sectioned and histologically examined by trained and board-certified surgical pathologists. Date and cause of death were obtained from the Epidemiological Cancer Registry of the State of Schleswig–Holstein, Germany, distinguishing between tumor-related deaths and deaths from other causes. Follow-up and clinical data of all included patients were obtained from hospital records and from oncologists of cooperating hospitals and private practices. Patient survival times, i.e., overall survival (OS) and tumor specific survival (TSS), were calculated from the time of surgery.

All patients gave written informed consent for routine surgery and chemotherapy. All patient data were pseudonymized after inclusion in the study.

Histologic processing and classification, as well as materials and methods for immunohistochemistry and in situ hybridization, the assessment of the Claudin 18.2-, HER2-, PD-L1- and of the microsatellite instability (MSI) /DNA mismatch repair protein (MMR) status and the statistical analyses are described in detail in the online resource.

Tumor regression was assessed using the Becker regression score, as published by Becker et al. [4]. The Becker regression score is a widely used instrument, which has been thoroughly validated and proved to be of prognostic value in the past [5]. To this end, the entire former tumor bed was embedded in paraffin and centrally assessed by two independent board-certified surgical pathologists, in order to account for inter-observer variability. The evaluation was centralized at the Department of Pathology at the University Hospital of Schleswig–Holstein in Kiel, Germany. The results of each assessment were obtained from the patients' histopathology reports. A Becker regression score of 1a, 1b, 2 or 3 were assigned, if no residual tumor/tumor bed, < 10% residual tumor/tumor bed, 10–50% residual tumor/tumor bed or > 50% residual tumor/tumor bed were seen, respectively [4].

## Results

### Study cohort

**Fig. 1:**
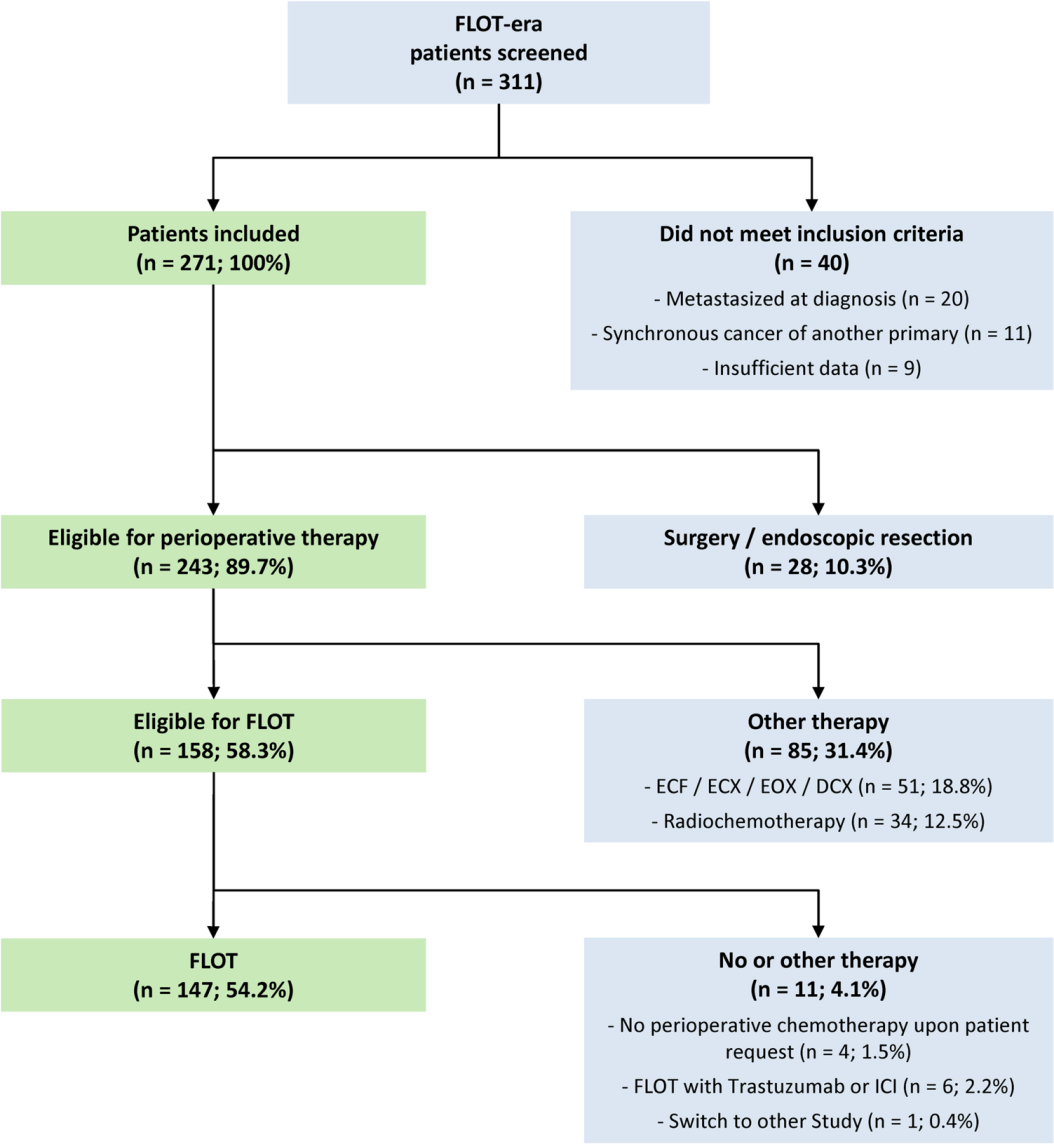
Patients screening. Description of the patient screening process. We screened 311 patients of the FLOT era and identified 147 patients who had received perioperative chemotherapy with FLOT

Three hundred and eleven patients were retrieved from the FLOT chemotherapy era (Fig. 1). 243 (89.7%) patients were eligible for perioperative therapy, of which 85 (31.4%) formed a heterogeneous treatment group and had not received FLOT. 158 (58.3%) patients were eligible for the FLOT regimen. However, 147 (54.2%) of the initial 271 potentially curable patients met all inclusion criteria and received at least one chemotherapy cycle of the FLOT regimen (Table 1). Our patients were predominantly male and frequently had proximally localized tumors, reflecting epidemiologic trends [6, 7].

**Table 1:** Base line characteristics of the study cohort

		*N*	(%)
Total		147	(100)
Sex	*N*/missing	147/0	
Male		120	(81.6)
Female		27	(18.4)
Age group	*N*/missing	147/0	
< 60		44	(29.9)
60–69		46	(31.3)
> = 70		57	(38.8)
Localization	*N*/missing	147/0	
AEG		121	(82.3)
Stomach		26	(17.7)
Lauren	*N*/missing	147/0	
Complete regression		16	(10.9)
Intestinal		59	(40.1)
Diffuse		24	(16.3)
Mixed		25	(17.0)
Unclassifiable		23	(15.6)
ypT	*N*/missing	147/0	
T0		17	(11.6)
T1a/T1b		14	(9.5)
T2		19	(12.9)
T3		89	(60.5)
T4a/T4b		8	(5.4)
ypN	*N*/missing	147/0	
N0		64	(43.5)
N1		28	(19.0)
N2		33	(22.4)
N3a/b		22	(15.0)
M	*N*/missing	147/0	
M0		147	(100.0)
M1		0	(0.0)
UICC stage	*N*/missing	147/0	
0		14	(9.5)
IA/B		25	(17.0)
IIA/B		32	(21.8)
IIIA/B/C		67	(45.6)
IV		9	(6.1)
Lymph node ratio	*N*/missing	147/0	
Median		0.048	
pL	*N*/missing	144/3	
L0		91	(63.2)
L1		53	(36.8)
pV	*N*/missing	143/4	
V0		130	(90.9)
V1		13	(9.1)
Pn	*N*/missing	137/10	
Pn0		93	(67.9)
Pn1		44	(32.1)
pR	*N*/missing	145/2	
R0		131	(90.3)
R1/R2		14	(9.7)
Tumor regression grade (Becker)	*N*/missing	144/3	
1a		17	(11.8)
1b		24	(28.5)
2		31	(21.5)
3		72	(50.0)
EBV status	*N*/missing	104/43	
Negative		102	(98.1)
Positive		2	(1.9)
MSI/MMR status	*N*/missing	124/23	
MSS/pMMR		120	(96.8)
MSI/dMMR		4	(3.2)
HER2 status	*N*/missing	130/17	
Negative		116	(89.2)
Positive		14	(10.8)
Surgery type	*N*/missing	147/0	
Open surgical procedure		44	(22.9)
Laparoscopic		8	(5.4)
Robotic		95	(64.6)
Overall survival [months]	*N*/missing	146/1	
Total/events/censored		146/89/57	
Median survival		19.4 ± 2.9	
95% C.I		[13.6–25.1]	
Tumor specific survival [months]	*N*/missing	132/15	
Total/events/censored		132/65/67	
Median survival		19.9 ± 3.1	
95% C.I		[13.7–26.0]	

*pL* lymphovascular invasion; *pV* vascular invasion; *pN* nodal status of the resection specimens; *MSI* microsatellite instability; *MMR* mismatch repair protein status; *dMMR* mismatch repair protein deficient; *pMMR* mismatch repair protein proficient

### Perioperative chemotherapy with FLOT

**Fig. 2:**
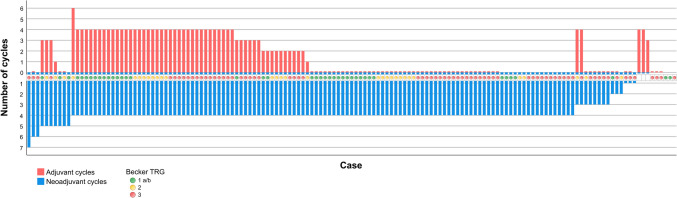
Waterfall plot depicting the neoadjuvant (blue bars) and adjuvant (red bars). Administration of perioperative chemotherapy with FLOT in the real-world study cohort. Indicated blue bar = patient received no neoadjuvant chemotherapy. Indicated red bar = patient received no adjuvant chemotherapy. No indicated red bar = no patient data about the adjuvant phase available. No indicated blue bar = no patient data about the neoadjuvant phase available. The tumor regression grades (TRG) are highlighted with colors, with TRG1, TRG2, TRG3 being represented by the colors green, yellow and red, respectively. Complete data about the neoadjuvant phase was available from 141 (95.9%) of 147 patients of the FLOT group. As three patients had not received neoadjuvant chemotherapy, the TRG was not applicable for these cases

One hundred forty-seven (now reported as 100%) patients were treated according to the FLOT-protocol between 2010 and 2022 (Fig. 2). Complete neoadjuvant and adjuvant chemotherapy data were available for 119 (81.0%) patients: 36 (24.5%) patients completed all four neoadjuvant and all four adjuvant cycles, 124 (84.4%) patients completed all four (or more) neoadjuvant cycles. Use of adjuvant chemotherapy was limited: Only 40 (27.2%) patients completed all four adjuvant cycles (Fig. 2). 59 (40.1%) patients had not received adjuvant chemotherapy. Details of perioperative chemotherapy administration, reasons for deviations and dose reductions are provided in the Online Resource Tables 1, 2, 3 and 4.

### Surgery and histology of resection specimens

Our patients underwent total or partial gastrectomy or esophagectomy, depending on the tumor site. Type of surgery (Table 1) was not associated with patient survival (open surgery versus laparoscopic/robotic surgery: OS 19.9 months versus 19.4 months, *p* = 0.408; TSS: 19.9 months versus 20.0 months, *p* = 0.587). Most of the surgeries were performed robotically. Data about the number of resected lymph nodes were available from all patients. The median numbers of lymph nodes resected were 27.0 for the entire cohort and 28.0, 23.5, 29.0 and 28.0 for the TRG1a, TRG1b, TRG2 and TRG3 subgroups, respectively. The type of surgery was not associated with the number of lymph nodes resected (median 28 versus 27 resected lymph nodes in open surgery versus laparoscopic/robotic surgery, *p* = 0.676 for Mann-Whitey-U test). The R0-resection rate was 90.3% (Table 1).

The majority of tumors had invaded the subserosa (ypT3; 60.5%), had lymph node metastases (56.5%) and were in UICC stage III and showed no signs of tumor regression (TRG3; 50.0%) (Table 1; Fig. 2). The number of EBV- or MSI-positive tumors was low.

### Survival analyses

The median follow-up of our patients was 45.4 months (95% CI, 25.9–64.8 months) with median OS and TSS of 19.4 (± 2.9) and 19.9 (± 3.1) months, respectively (Table 1). The 30-day postoperative mortality rate was 1.38%. The 2-year OS for the overall cohort was 39.3% (± 4.9%) and the 2-year TSS was 44.3% (± 5.5%). The 5-year OS was 12.7% (± 3.9%) and the 5-year TSS was 18.5% (± 5.3%).

**Fig. 3:**
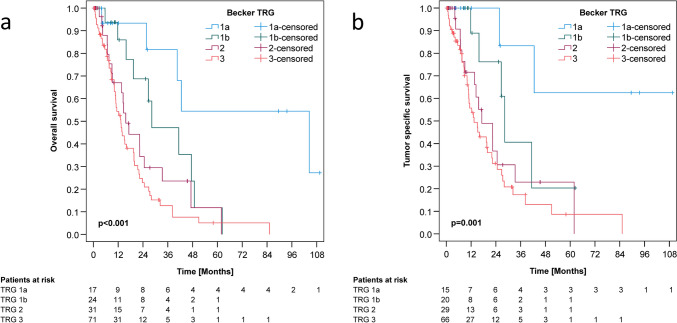
Kaplan–Meier estimates of overall (**a**) and tumor-specific (**b**) survivals. **a**, **b**: Comparison of histopathologic tumor regression (TRG) according to Becker

**Fig. 4:**
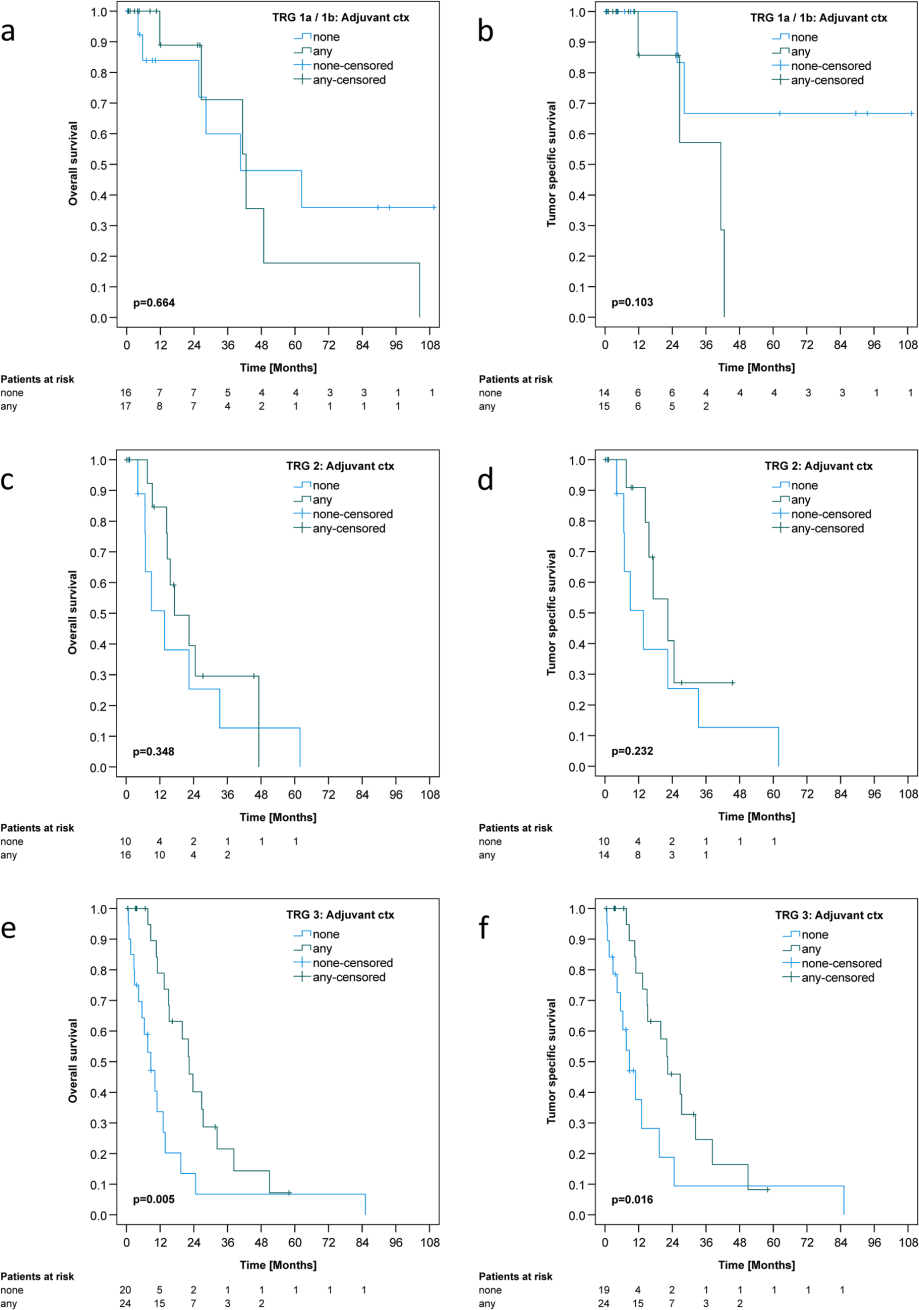
Kaplan–Meier estimates of overall (**a**, **c**, **e**) and tumor-specific (**b**, **d**, **f**) survivals. **a**, **b**: Comparison of adjuvant chemotherapy versus no adjuvant chemotherapy in patients with TRG1a/b. **c**, **d**: Comparison of adjuvant chemotherapy versus no adjuvant chemotherapy in patients with TRG2. **e**, **f**: Comparison of adjuvant chemotherapy versus no adjuvant chemotherapy in patients with TRG3

In univariate analysis, ypT category, ypN category, lymph node ratio, lymphovascular invasion, venous invasion, resection status, and TRG (Figs. 3 and 4) correlated with both, OS and TSS, respectively. The age group correlated only with TSS (Online Resource Table 5). In multivariate analyses, age group, ypT category, venous invasion, and resection status remained independent predictors of OS, whereas age group, venous invasion, the resection status and TRG were independent predictors of TSS (Online Resource Table 5). To compensate for the false discovery rate within statistical tests, we applied the Siemes (Benjamini-Hochberg) procedure, which identified only the p-values of the multivariate TRG survival analyses (overall TRG1a vs. TRG1b vs. TRG2 vs. TRG3) that would be lost by correction for multiple testing. The remaining correlations remained significant (Online Resource Table 5), explicitly including the results found for the subsequent analyses of adjuvant chemotherapy administration in relation to TRG status (see below for details).

We then evaluated the effect of the number of FLOT cycles on patient outcomes. No difference was found between patients who completed all cycles of perioperative FLOT and those who did not (OS *p* = 0.560; TSS *p* = 0.893). There was also no difference when all four neoadjuvant cycles were administered compared to less than four cycles (OS *p* = 0.958; TSS *p* = 0.219).

Next, we correlated perioperative chemotherapy and patient outcome according to TRG categories (Figs. 3 A, B; 4 A–F). Notably, only patients who had received at least all four cycles of neoadjuvant chemotherapy were included in the analysis of adjuvant chemotherapy efficacy (Fig. 2): Remarkably, neoadjuvantly treated patients with TRG1 (TRG1a complete histopathologic remission; TRG1b < 10% residual tumor mass) did not benefit from any number of adjuvant chemotherapy cycles (OS *p* = 0.664; TSS *p* = 0.103) (Fig. 4A, B). Patients with a partial (TRG2) histopathologic response after four cycles of neoadjuvant chemotherapy showed a strong trend toward longer OS and TSS [(OS: 17.2 ± 5.1 months versus 13.7 ± 4.6 months; *p* = 0.348); TSS (22.4 ± 4.3 months versus 13.7 ± 4.6 months; *p* = 0.232)], without reaching statistical significance (Fig. 4C, D).

Patients with minimal/no histopathologic response (TRG3) after at least four cycles of neoadjuvant chemotherapy lived significantly longer when any number of adjuvant chemotherapy cycles were administered [(OS: 22.3 ± 2.6 months versus 8.7 ± 2.4 months; *p* = 0.005); TSS (22.3 ± 4.5 months versus 8.7 ± 2.4 months; *p* = 0.016)] (Fig. 4E, F). Also, Cox regression for this subgroup showed a hazard ratio for OS of 2.693 and for TSS a hazard ratio of 2.502 for having received no adjuvant chemotherapy versus having received at least one cycle of adjuvant chemotherapy (OS: *p* = 0.007, 95% confidence interval 1.318–5.501; TSS: *p* = 0.019, 95% confidence interval 1.163–5.379). The effect was independent of individual dose reductions or omissions of chemotherapeutic agents. The results remained significant after correction for multiple testing. The survival analysis of the TRG3 subgroup is further supported by the results of the post-hoc power analysis, which is described in detail in the online resource.

The median time between surgery and initiation of adjuvant chemotherapy was 49 days (range 28–142 days) (Online Resource Fig. 1).

The number of MSI- and EBV-positive tumors and the documentation of the Eastern Cooperative Oncology Group (ECOG) performance status was too low for statistical analysis (data not shown).

### Biomarker profile of the TRG2/3 subgroup

**Fig. 5:**
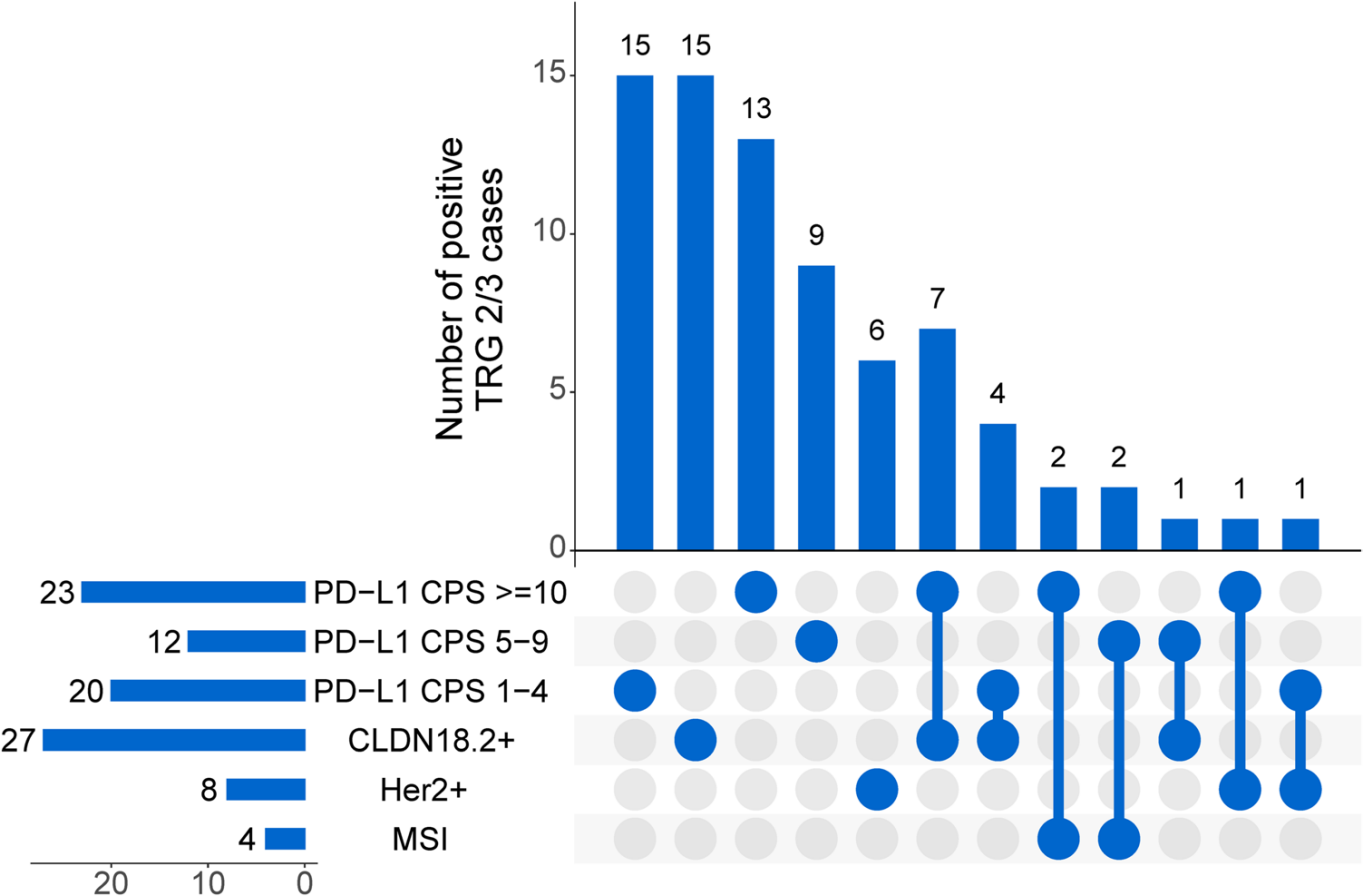
Distribution of targetable biomarkers in the TRG2/3 patient subgroup. UpSet plot showing the distribution of the targetable biomarkers PD-L1, Claudin 18.2 and HER2 as well as the MSI (microsatellite instability) status of the 103 TRG2/3 patients. PD-L1 expression with combined positive scores (CPS) of 1–4, 5–9 and ≥ 10 were observed in 20 (19.4%), 12 (11.7%) and 23 (22.3%) patients, respectively. The biomarkers HER2 and Claudin 18.2 were expressed in 8 (7.8%) and 27 (26.2%) patients of the TRG2/3 patient subset, respectively. We identified 4 patients (3.9%) with MSI tumors. Biomarker co-expression is visualized with connecting bars and enumerated in the upper part of the illustration

The TRG2/3 subset included 103 patients. The biomarker status of PD-L1, Claudin 18.2, HER2 and MSI was positive in 55 (53.4%), 27 (26.2%) and 8 (7.8%) of these patients, respectively. Specifically, PD-L1 expression with combined positive scores (CPS) of 1–4, 5–9 and ≥ 10 was observed in 20 (19.4%), 12 (11.7%) and 23 (22.3%) patients, respectively. We identified 4 patients (3.9%) with MSI tumors. Biomarker co-expression was reported in 17.5% of the subset. As summarized in the UpSet plot (Fig. 5), 73.8% of patients with TRG2/3 expressed one of the biomarkers investigated.

## Discussion

Our study underscores the crucial role of the adjuvant phase of the FLOT regimen in a real-world setting. The survival rates of our patients were surprisingly low. The 5-year survival rate was even lower than that of the perioperative chemotherapy arm of the now historic MAGIC trial [2]. We performed a dedicated analysis in order to identify and rule out factors that could have led to such unfavorable survival rates:

First, we found that the neoadjuvant phase in our cohort was implemented with competitive efficiency and cannot be used to explain the lack of survival benefit: The pathological complete response (pCR) rate in our study was 11.8%, which is comparable to the pCR of the FLOT control arms of several contemporary chemotherapy trials, including the MATTERHORN trial [8], or the DANTE Phase II trial [9]. Our real-world analysis showed that 84.4% of all patients had completed at least four cycles of neoadjuvant chemotherapy, which is only slightly lower than the 90% rate observed in the neoadjuvant phase of the original FLOT4 trial [3] or the 95% rate observed in the neoadjuvant phase of the FLOT control arm of the DANTE trial [10]. We further show that surgical procedures cannot be used to explain the unfavorable survival rates. The 30-day postoperative survival rates were even better than the 30-day survival rates reported in the FLOT4 trial [3], the median number of lymph nodes resected was comparable to that reported in the recently published SPACE-FLOT study [11] and the R0 resection rates were even slightly higher (90.3% versus 88.6%) than those reported in the SPACE-FLOT study [11].

Our data underscore that the implementation of adjuvant chemotherapy in daily clinical practice represents an obvious obstacle in the real world. Should all patients receive adjuvant chemotherapy whenever possible, and if not, which subgroup should be targeted?

We hypothesized that the omission of adjuvant chemotherapy would affect patient survival. Surprisingly, however, at first glance there was no significant difference in survival between patients with and without adjuvant chemotherapy, which was started in only 42.9% and completed in only 27.2% of our patients.

Although a correlation between TRG and patient prognosis in the perioperative setting has been frequently described, its prognostic value is still debated due to different scoring systems with different cut-off values complicated by inter- and intra-observer variability [5]. In the past, we have confirmed superior survival rates for patients with < 10% tumor regression using digital image analysis, a cut-off used in the Becker TRG scoring system [5]. A central question of our current study was how the continuation or omission of adjuvant chemotherapy would affect real-world outcomes depending on the degree of tumor regression after neoadjuvant chemotherapy. Real-world studies from the time before or during the transition to the FLOT era paint an ambiguous picture in this regard: In their study from the pre-FLOT era, Deng et al. described that complete and non-responders to neoadjuvant chemotherapy did not benefit from adjuvant chemotherapy, based on a comparison between T stage at diagnosis and yT stage of the resection specimen after neoadjuvant chemotherapy [12]. Saunders et al. described in their study from the pre-FLOT era that adjuvant therapy would only benefit those patients, who showed a histopathological response after neoadjuvant chemotherapy and based their findings on the old Mandard TRG scoring system [13]. In contrast, the study by Glatz et al., which analyzed a mixed population receiving the now historic chemotherapy regimens ECF and EOX, but also the FLOT regimen, described that patients with poor histopathologic regression according to Becker et al. (TRG 3) benefited from adjuvant chemotherapy [14]. To address this highly relevant therapeutic issue in a contemporary context, real-world studies involving only patients receiving the current chemotherapy regimen with FLOT are needed.

Our real-world study included only patients who had received the current FLOT regimen. We analyzed whether the effect of adjuvant chemotherapy on survival depended on tumor regression and restricted our analysis to those patients who had received at least all four neoadjuvant chemotherapy cycles. We found that adjuvant chemotherapy did not improve survival in TRG1 patients. For TRG2 patients, there was a strong trend toward longer survival with any number of adjuvant chemotherapy cycles, but this trend did not reach statistical significance. However, TRG3 patients lived significantly longer with any number of adjuvant chemotherapy cycles, regardless of dose reductions or the omission of chemotherapeutic drugs.

Our findings are partially supported by the results of the recently published retrospective SPACE-FLOT study [11], which confirms a key finding of our study: lack of benefit of adjuvant chemotherapy in patients with complete tumor regression. Furthermore, they described a significant benefit of adjuvant chemotherapy in patients with a partial histopathologic response, which supports the strong trend for longer survival seen in our collective for TRG2 patients. However, in the SPACE-FLOT study population, patients with minimal or no tumor regression (TRG3) did not benefit from adjuvant chemotherapy, which is in contrast to our findings.

German medical centers did not participate in the SPACE-FLOT study, but we do not believe that geographic differences were responsible for the different results regarding the subgroup of patients with minimal or no tumor regression. Furthermore, we do not believe that the different results are due to the concepts of TRG evaluation. In our study, TRG evaluation was centrally performed by two independent board-certified surgical pathologists to reduce interobserver variability, whereas in the SPACE-FLOT study TRG was not centrally assessed [11]. Equivalent to the international Delphi consensus [15] used in the SPACE-FLOT study, in our study the entire tumor was always embedded and analyzed. While we consistently used the Becker scoring system, the SPACE-FLOT cohort used seven different TRG systems. A practical approach was needed to unify these systems for data analysis [11]. Nevertheless, we believe that with respect to the definition of minimal/no tumor regression the TRG scoring between our study and the SPACE-FLOT study is comparable, as they defined minimal/no tumor regression as the presence of > 50% residual tumor in the resection specimen, which is equivalent to TRG3 according to Becker et al.

We believe that the relevant discrepancy regarding the effect of adjuvant chemotherapy on survival of patients with minimal/no tumor regression may be explained by different approaches to the analysis of adjuvant chemotherapy efficacy and by different R0 resection rates.

In our study, only patients who had received at least all four neoadjuvant chemotherapy cycles, and thus had received optimal neoadjuvant treatment, were included in the subsequent survival analysis related to TRG and adjuvant chemotherapy administration. In the SPACE-FLOT patient subgroup with minimal or no histopathologic response, 87.9% of those with adjuvant treatment and only 66.8% of those without adjuvant treatment had received all four cycles of neoadjuvant chemotherapy. Although the rationale of neoadjuvant chemotherapy with FLOT is the reduction of tumor size for surgery, it is conceivable that it could also influence metastatic spread, relapse and survival. Incomplete administration of neoadjuvant chemotherapy, whether due to internal or external factors, may greatly affect the histopathologic response and the conclusions drawn from it.

The R0 resection rate in our study cohort was high at 90.3% overall and 85.9% in the subgroup with minimal or no tumor regression (100% in the subgroup that received adjuvant chemotherapy and 90.0% in the subgroup that did not receive adjuvant chemotherapy with FLOT). However, in the SPACE-FLOT trial, the R0 resection rate in the subgroup with minimal or no tumor regression was only 82.7% for patients who received adjuvant chemotherapy and 76.5% for patients who did not receive adjuvant treatment. Therefore, in the SPACE-FLOT trial, adjuvant chemotherapy had to compensate for considerably more patients in whom complete surgical removal of the cancer site was not possible than in our study cohort, in which considerably better surgical results were achieved. In addition, because in the SPACE-FLOT trial the R0 resection rates were comparatively low in both subgroups with or without adjuvant chemotherapy, no statistical difference was observed in the univariate analysis and we assume that the resection rate was therefore not considered in the subsequent Cox regression analysis and propensity score matching analysis of the trial. We postulate that in the case of patients with minimal or no tumor regression after neoadjuvant chemotherapy, adjuvant chemotherapy cannot compensate for incomplete surgical resection of the cancer site because the poorly responding primary tumor is still present in its original setting.

The focus of adjuvant chemotherapy is to eliminate occult distant metastases, which have a different tumor microenvironment than the primary tumor site. In the past we have shown that adjuvant chemotherapy in the perioperative setting addresses a completely different tumor [5, 16, 17]. This differs from the current concept of adjuvant chemotherapy in the FLOT era and the conclusions drawn by the SPACE-FLOT trial. We believe that a minimal or poor histopathologic response at the primary site is not predictive of chemotherapy efficacy at the metastatic site, as a completely different tumor environment is being targeted. The data presented here demonstrate that the TRG3 subset of patients benefited from adjuvant treatment and we postulate that this effect was seen, in contrast to the SPACE-FLOT trial, because the R0 resection rate in our cohort was high and the effect of adjuvant chemotherapy with FLOT in our cohort reflects its effect on the elimination of peripheral micrometastases rather than on residual tumor cells at the primary tumor site.

The subgroup of patients with partial or no response to neoadjuvant chemotherapy with FLOT have an overall poor prognosis and should be the focus of future therapeutic efforts. Our study therefore provided a dedicated biomarker analysis that could serve as a basis for the design of future randomized controlled trials. We see a need for personalization of the adjuvant regimen based on the biomarker profile of the resected tissue specimen—not the initial biopsies. Patients with partial or no histopathologic response may benefit from the addition of targeted therapies to the chemotherapy backbone in the adjuvant setting. Recently, the results of the VESTIGE trial were published, which evaluated adjuvant immunotherapy in patients with resected GC or AEG following preoperative chemotherapy and high risk for recurrence (ypN + and/or R1 status) [18]. The chemotherapy-free approach had not been successful in this high-risk subset of patients [18], thereby underscoring the role of the chemotherapy backbone. Future studies are needed to investigate the potential benefits of adding targeted therapy to the chemotherapy backbone in the adjuvant setting.

### Limitations

The limitation of our study is its retrospective nature and of course this is only a cohort of patients who underwent tumor surgery. As comorbidities naturally increase with age, an assessment of comorbidities would have been desirable and should be included in future real-world studies. The ECOG performance status was not regularly documented in our retrospective cohort, which limits our results in this regard. In addition, we believe that the relatively small size of the TRG2 subgroup resulted in the observation of only a strong trend and not a significant survival benefit for TRG2 patients receiving adjuvant chemotherapy as seen in the SPACE-FLOT trial.

## Conclusions

In conclusion, the adjuvant phase proved to be the critical turning point for the clinical implementation of the FLOT regimen in the real-world setting. Our results suggest that adjuvant chemotherapy with FLOT—even in a reduced form—should be sought in patients with TRG2/3, but may be unnecessary in patients with TRG1.

Our findings will inform the design of future clinical studies aimed at improving adjuvant gastric cancer therapy in TRG2/3 patients through the implementation of personalized treatment regimens based on the biomarker profile of the resected tissue specimens.

## Supplementary Information

Below is the link to the electronic supplementary material.

Supplementary file1 (PDF 328 KB)

## Data Availability

The datasets (deidentified participant data) generated during and/or analyzed during the current study are available from the corresponding author on reasonable request for researchers who provide a methodologically sound proposal following the publication of the manuscript. The proposal has to be approved by Prof. Dr. med. Christoph Roecken and the local ethics committee of the University Hospital Schleswig–Holstein, Kiel, Germany. Data requestors will need to sign a data access agreement. Proposals should be directed to christoph.roecken@uksh.de.
