# Supplementary material for: Impact of adjuvant therapy on outcomes of cancer of the stomach and gastroesophageal junction in the real-world
Source: Gastric Cancer. 2025 May 16;28(5):982–92. doi: 10.1007/s10120-025-01624-8 (PMC12378129; doi:10.1007/s10120-025-01624-8)
Supplement: Supplementary file 1 — Supplementary file1 (PDF 328 KB) [file 10120_2025_1624_MOESM1_ESM.pdf]

Supplementary Online Content

Impact of Adjuvant Therapy on Outcomes of Cancer of the Stomach and Gastroesophageal Junction in the Real-World

Steffen M. Heckl, Hans-Michael Behrens*, Ulrike Ebert*, Dita Ulase, Florian Richter, Thomas Becker, Anne Letsch, Christoph Röcken[1]

*Both authors contributed equally to this work.

[1]Corresponding author: Christoph Röcken; christoph.roecken@uksh.de; Department of Pathology, Christian-Albrechts-University, University Hospital Schleswig-Holstein, Kiel, Germany

Supplementary Methods

Supplementary Results

Supplementary Table 1. Preoperative setting.

Supplementary Table 2. Reasons for a reduction of neoadjuvant chemotherapy cycles.

Supplementary Table 3. Postoperative setting.

Supplementary Table 4. Reasons for omission of adjuvant chemotherapy.

Supplementary Table 5. Univariate and multivariate analysis.

Supplementary Figure 1. Time intervals between surgery and adjuvant chemotherapy initiation and survival.

Post-hoc power calculation

References

Supplementary methods

Histology

Tissue specimens were fixed in formalin and embedded in paraffin. Deparaffinized sections were stained with hematoxylin and eosin. Histological re-examination of primary tissue sections was carried out for all cases to ensure that inclusion criteria were met. Tumors were classified according to the Laurén classification (1) and re-examined by two surgical pathologists. pTNM-stage or ypTNM-stage of all study patients was determined according to the 8th edition of the UICC guidelines (2). For further clarification we would like to highlight an important peculiarity in the context of the UICC staging system for gastric cancer (GC) and for cancer of the esophagogastric junction (AEG): In the case of the UICC classification of GC, a UICC IV stage indicates the presence of distant metastasis, which was an exclusion criterion for our study. According to the UICC classification of cancers of the esophagus, a UICC IVa stage is given if a cancer of the esophago-gastric junction has a pT4b stage (and N0 or N1 or N2) or a N3 stage without the presence of distant metastasis and hence an inclusion of the respective patients was possible despite the UICC IV stage.

The Becker regression score (3) was used to assess tumor regression and is described in detail in the main manuscript.

Immunohistochemistry and *in situ* hybridization

Immunohistochemistry was carried out with monoclonal antibodies directed against Her2/neu (dilution 1:200; Thermo Fisher Scientific, Fermont, USA), MLH1 (dilution 1:20; mouse monoclonal; BD Pharmingen, San Diego, USA), PMS2 (dilution 1:20; mouse monoclonal; Cell Marque, Rocklin, CA, USA), MSH2 (dilution 1:100; mouse monoclonal; Calbiochem, San Diego, USA), MSH6 (dilution 1:50; mouse monoclonal, BD Transduction Laboratories, BD Biosciences, San Jose, CA, USA), PD-L1 and claudin 18.2 (clone 43-14A, Abcam) on the autostainer BondTM Max System (Leica-Menarini, Berlin, Germany). Antigen retrieval was performed using the ER2 antigen retrieval solution for 20 min at pH 9.0 (Leica-Menarini) for MLH1, PMS2, and MSH2. For MSH6, antigen retrieval was carried out using the ER1 antigen retrieval solution for 20min at pH 6.0 (Leica-Menarini) and for MSH6 the DAB Enhancer (Leica-Menarini) was employed to enhance the immunostaining. For chromogenic *in situ* hybridization the ZytoDot® 2C SPEC ERB2/CEN 17 Probe Kit (ZytoVision GmbH, Bremerhaven, Germany) was used.

Assessment of the HER2 status

The assessment of the HER2-status was performed as previously described (4). In brief, the immunostaining intensity was evaluated and scored ranging from negative (0) to strong (3+). For all GC / AEG cases with an immunostaining of 2+, a chromogenic *in situ* hybridization was performed. If a moderate immunostaining (2+) together with a HER2 gene amplification (ratio ≥2.0) or a strong immunostaining (3+) was present in ≥10% of the tumor area, the sample was classified as HER2-positive.

Assessment of claudin 18.2 status

The Claudin 18.2 status was determined according to the Spotlight study. In brief: a GC was considered positive when ≥75% of tumor cells showed moderate to strong membranous expression (5).

Assessment of the microsatellite instability status

The microsatellite instability (MSI) / DNA mismatch repair protein (MMR) status was assessed as previously published elsewhere (6).

Quality Assurance

The University Hospital Schleswig-Holstein Campus Kiel is an Oncology Centre that was initially certified by the German Cancer Society in 2013. Since that time, the center has successfully undergone annual re-certification.

External Quality Assurance

The immunohistochemical evaluation of DNA mismatch repair proteins (MSH2, MSH6, MLH1 and PMS2), the molecular pathological evaluation of MSI and the evaluation of HER2 and PD-L1 status in gastric cancer have been successfully certified by the quality assurance program of the German Society of Pathology and the Bundesverband Deutscher Pathologen e.V. (for more details see https://www.patho.uni-kiel.de/krankenversorgung/qualitaetssicherung).

Statistical analyses

SPSS version 27 (IBM Corp., Armonk, NY, USA) and R Statistical Software version 4.4.0 (R Core Team) were used for statistical analyses. Age, T category, N category, and UICC stage as ordinal scale variables were tested with Kendall's tau test. Median survival with 95% confidence intervals was calculated using the Kaplan-Meier method. Differences between median survival times were tested with the log-rank test. Correlations between TRG and patient age groups were calculated with the test for equality of proportions using R.

Multivariate survival analysis (Cox regression) was performed with SPSS using the backward-LR method. A p-value ≤ 0.05 was considered significant. All p-values are uncorrected. To compensate for the false discovery rate within statistical tests, we applied the Siemes (Benjamini-Hochberg) procedure (7). P-values that lost significance are highlighted.

The post-hoc power analysis is described separately at the end of the supplement.

Supplementary results

Supplementary Table 1. Preoperative setting.

Complete data were available from 141 (95.9%) of 147 patients of the FLOT group.

Neoadjuvant treatment	n (%)
More than four neoadjuvant FLOT cycles*	10 (6.8)
Four neoadjuvant FLOT cycles	114 (77.6)
Three neoadjuvant FLOT cycles	8 (5.5)
Two neoadjuvant FLOT cycles	3 (2.0)
One neoadjuvant FLOT cycle	3 (2.0)
No neoadjuvant FLOT cycles**	3 (2.0)
Total number of cases with complete data	141 (95.9)

* In five patients, the individual decision to opt for more neoadjuvant cycles was based upon the treating physicians' conceptualization about being able to reduce the number of adjuvant chemotherapy cycles in return. In the remaining five cases the rational could not be retrieved from the database.
**In one patient the histopathological analysis of the tumor specimen following primary resection had shown lymph node metastasis and therefore an understaging. For the second patient, it had not been possible to confirm the cancer diagnosis via endoscopic biopsies and therefore the decision had been made to perform a primary surgical resection without neoadjuvant chemotherapy. A third patient received a primary surgery due to a previous understaging and therefore only received adjuvant chemotherapy.

Supplementary Table 2. Reasons for a reduction of neoadjuvant chemotherapy*

Reason	n
Tumor progression	2
Domestic care problems	1
Serious tumor bleeding occurred – earlier surgery necessary	1
Port thrombosis / thrombosis of the V. jugularis interna and replacement of the intravenous 5-FU with capecitabine	1
Limited tolerability of the chemotherapy regimen accompanied by a successive deterioration of the patients' general health	4
Unspecific thoracic complaints with the presumptive diagnosis of a 5-FU induced angina pectoris	1
Severe colitis with clostridium difficile	1
Reasons unknown	3
Total	14

*Data about dose reductions during the neoadjuvant phase were available for 100 patients. Dose reductions were necessary during the neoadjuvant phase in 36 (24.5%) patients. 39 (26.5%) patients were subject to either a reduction of chemotherapy cycles or dose reductions during the neoadjuvant chemotherapy phase.

Supplementary Table 3. Postoperative setting.

Complete data were available from 122 (83.0%) out of 147 patients of the FLOT group*.

**Adjuvant treatment**	**n (%)**
Four adjuvant FLOT cycles	40 (27.2)
Four neoadjuvant FLOT cycles + 6 cycles of adjuvant chemotherapy with Capecitabin/Oxaliplatin**	1 (0.7)
Three adjuvant FLOT cycles	10 (6.8)
Two adjuvant FLOT cycles	10 (6.8)
One adjuvant FLOT cycle	2 (1.4)
No adjuvant FLOT cycles	59 (40.1)
Unknown, if the recommended adjuvant chemotherapy had been administered, as the respective data were not accessible	25 (17.0)
Total	147 (100.0)

*Data about dose reductions during the adjuvant phase were available for 121 patients. Dose reductions were necessary during the adjuvant phase in 43 (29.3%) patients. 104 (70.7%) of all patients were subject to either a reduction of chemotherapy cycles or dose reductions during the adjuvant chemotherapy phase. There were no differences with respect to reductions of dosage (p=1.000) or the number of adjuvant chemotherapy cycles (p=0.850) between women and men.
**reason for choice of chemotherapy regimen unknown.

Supplementary Table 4. Reasons for omission of adjuvant chemotherapy.*

**Reason**	**n (%)**
Postoperative complications	13 (8.8)
Patient choice	6 (4.1)
Previous chemotherapy toxicity	3 (2.0)
Postoperative death	5 (3.4)
Diagnosis of a myelodysplastic syndrome with pancytopenia	1 (0.7)
pR1 resection and metastatic spread before start of adjuvant chemotherapy	1 (0.7)
Progression between end of neoadjuvant therapy and surgery	1 (0.7)
Stroke	1 (0.7)
Unknown	28 (19.0)
Total	59 (40.1)

*Five patients with pR1 resection received adjuvant radiochemotherapy instead of a FLOT-protocol. In one patient with pR1 resection, radiotherapy was combined with adjuvant FLOT regimen.
In a second patient a switch to radiochemotherapy was made after administration of two rounds of FLOT as an individual approach for an R0 (CRM+) resection status.

Supplementary Table 5. Univariate and multivariate survival analysis.

		Overall survival (OS)								Tumor specific survival (TSS)							
		Univariate survival analysis (Kaplan-Meier)				Multivariate survival analysis (Cox regression)				Univariate survival analysis (Kaplan-Meier)				Multivariate survival analysis (Cox regression)			
		N total / events / censored	Median survival ± Std error [months]	95% CI for median survival [months]	p-value of log-rank test	Compared levels	Hazard ratio (HR)	95% CI for HR	p-value of Cox regression	N total / events / censored	Median survival ± Std error [months]	95% CI for median survival [months]	p-value of log-rank test	Compared levels	Hazard ratio (HR)	95% CI for HR	p-value of Cox regression
**N Total / events / censored**		146 / 89 / 57				127 / 85 / 42				132 / 65 / 67				114 / 63 / 51			
**Age group**	**n / missing**	146 / 89 / 57			0.075				0.002	132 / 65 / 67			0.018				0.001
< 60		44 / 26 / 18	19.6 ± 7.9	4.1 - 35.2		---				40 / 20 / 20	27.3 ± 7.5	12.6 - 42.0		---			
60 - 69		45 / 23 / 22	22.0 ± 2.1	17.8 - 26.3		60-69 vs <60	0.808	0.435 - 1.501	0.500	41 / 15 / 26	26.8 ± 4.2	18.7 - 34.9		60-69 vs <60	0.704	0.334 - 1.481	0.355
>= 70		57 / 40 / 17	14.0 ± 2.8	8.4 - 19.5		>=70 vs <60	2.109	1.226 - 3.630	0.007	51 / 30 / 21	13.4 ± 3.2	7.1 - 19.7		>=70 vs <60	2.490	1.317 - 4.708	0.005
**pT**	**n / missing**	146 / 89 / 57			<0.001				0.005	132 / 65 / 67			<0.001				
T0		17 / 5 / 12	104.4 ± 33.1	39.4 - 169.4		---				15 / 2 / 13	nc	nc					
T1a / T1b		14 / 8 / 6	28.0 ± 11.9	4.8 - 51.3		T1 vs T0	3.474	0.888 - 13.594	0.074	13 / 5 / 8	28.0 ± 1.4	25.3 - 30.7					
T2		19 / 11 / 8	15.8 ± 1.9	12.0 - 19.6		T2 vs T0	7.788	1.995 - 30.406	0.003	15 / 7 / 8	15.8 ± 3.1	9.9 - 21.8					
T3		88 / 59 / 29	15.7 ± 3.0	9.7 - 21.6		T3 vs T0	8.461	2.470 - 28.987	<0.001	81 / 46 / 35	17.2 ± 3.0	11.3 - 23.0					
T4a / T4b		8 / 6 / 2	8.5 ± 5.3	0.0 - 18.8		T4 vs T0	10.800	2.139 - 54.541	0.004	8 / 5 / 3	13.1 ± 5.5	2.2 - 23.9					
**pN**	**n / missing**	146 / 89 / 57			<0.001					132 / 65 / 67			<0.001				
N0		64 / 25 / 39	28.0 ± 10.5	7.5 - 48.6						58 / 16 / 42	28.0 ± 11.8	4.8 - 51.2					
N1		28 / 20 / 8	16.0 ± 3.4	9.4 - 22.7						27 / 16 / 11	19.9 ± 4.1	11.7 - 28.0					
N2		32 / 27 / 5	17.2 ± 4.0	9.3 - 25.1						29 / 22 / 7	17.2 ± 4.4	8.5 - 25.9					
N3a/b		22 / 17 / 5	8.6 ± 1.6	5.5 - 11.8						18 / 11 / 7	8.6 ± 0.8	7.0 - 10.3					
**LN ratio**	**n / missing**	146 / 89 / 57			<0.001					132 / 65 / 67			<0.001				
low		73 / 30 / 43	28.0 ± 10.0	8.5 - 47.6						66 / 20 / 46	38.3 ± 11.2	16.3 - 60.3					
high		73 / 59 / 14	13.1 ± 1.6	9.9 - 16.2						66 / 45 / 21	13.7 ± 3.3	7.3 - 20.1					
**L**	**n / missing**	143 / 89 / 54			0.002					129 / 65 / 64			0.002				
L0		90 / 48 / 42	23.7 ± 1.5	20.7 - 26.7						83 / 34 / 49	24.6 ± 2.8	19.1 - 30.2					
L1		53 / 41 / 12	10.9 ± 2.4	6.3 - 15.5						46 / 31 / 15	10.8 ± 0.9	9.1 - 12.5					
**V**	**n / missing**	142 / 88 / 54			<0.001				<0.001	128 / 64 / 64			<0.001				<0.001
V0		129 / 75 / 54	21.5 ± 1.5	18.6 - 24.4		---				116 / 53 / 63	22.4 ± 3.0	16.5 - 28.3		---			
V1		13 / 13 / 0	6.6 ± 1.9	2.9 - 10.2		V1 vs V0	5.019	2.229 - 11.298	<0.001	12 / 11 / 1	6.8 ± 3.3	0.2 - 13.4		V1 vs V0	5.836	2.700 - 12.617	<0.001
**R**	**n / missing**	144 / 87 / 57			<0.001				<0.001	130 / 64 / 66			<0.001				<0.001
R0		130 / 75 / 55	21.5 ± 1.5	18.6 - 24.3		---				118 / 54 / 64	22.4 ± 3.0	16.5 - 28.3		---			
R1 / R2		14 / 12 / 2	7.6 ± 3.9	0.0 - 15.3		R1/R2 vs R0	3.482	1.774 - 6.836	<0.001	12 / 10 / 2	6.8 ± 2.4	2.0 - 11.6		R1/R2 vs R0	7.458	3.255 - 17.086	<0.001
**Becker TRG**	**n / missing**	143 / 88 / 55			<0.001					130 / 65 / 65			0.001				0.026*
1a		17 / 5 / 12	104.4 ± 33.1	39.4 - 169.4						15 / 2 / 13	nc	nc		---			
1b		24 / 10 / 14	28.3 ± 9.8	9.1 - 47.4						20 / 5 / 15	28.3 ± 1.7	25.0 - 31.5		1b vs 1a	1.753	0.315 - 9.760	0.522
2		31 / 19 / 12	15.7 ± 2.1	11.6 - 19.8						29 / 15 / 14	17.2 ± 6.3	4.8 - 29.6		2 vs 1a	6.246	1.320 - 29.547	0.021
3		71 / 54 / 17	13.3 ± 1.5	10.3 - 16.3						66 / 43 / 23	13.4 ± 2.6	8.4 - 18.5		3 vs 1a	4.637	1.021 - 21.068	0.047*

All variables with $P < 0.100$ in the univariate survival analyses (log-rank test, overall survival / tumor specific survival) are shown and were included in the subsequent multivariate survival analyses (Cox regression, overall survival / tumor specific survival). The results are separately shown for overall and for tumor specific survival. Upon multivariate analysis, patient age, ypT category, venous invasion and resection status were found to be independent prognosticators of overall patient survival. Patient age, venous invasion, resection status and tumor regression (TRG) were independent prognosticators of tumor specific survival. HR= Hazard ratio. nc: cannot be calculated because curve does not drop below 50% level. P-values that have lost significance according to the Siemes (Benjamini-Hochberg) multiple testing procedure are marked with an asterisk.

Supplementary Figure 1. Time intervals between surgery and adjuvant chemotherapy initiation and survival.

Histogram (A) depicting the distribution of time intervals between surgery and adjuvant chemotherapy initiation. Kaplan-Meier estimates of overall (B) and tumor-specific (C) survival as a function of the time interval between surgery and the start of adjuvant chemotherapy.

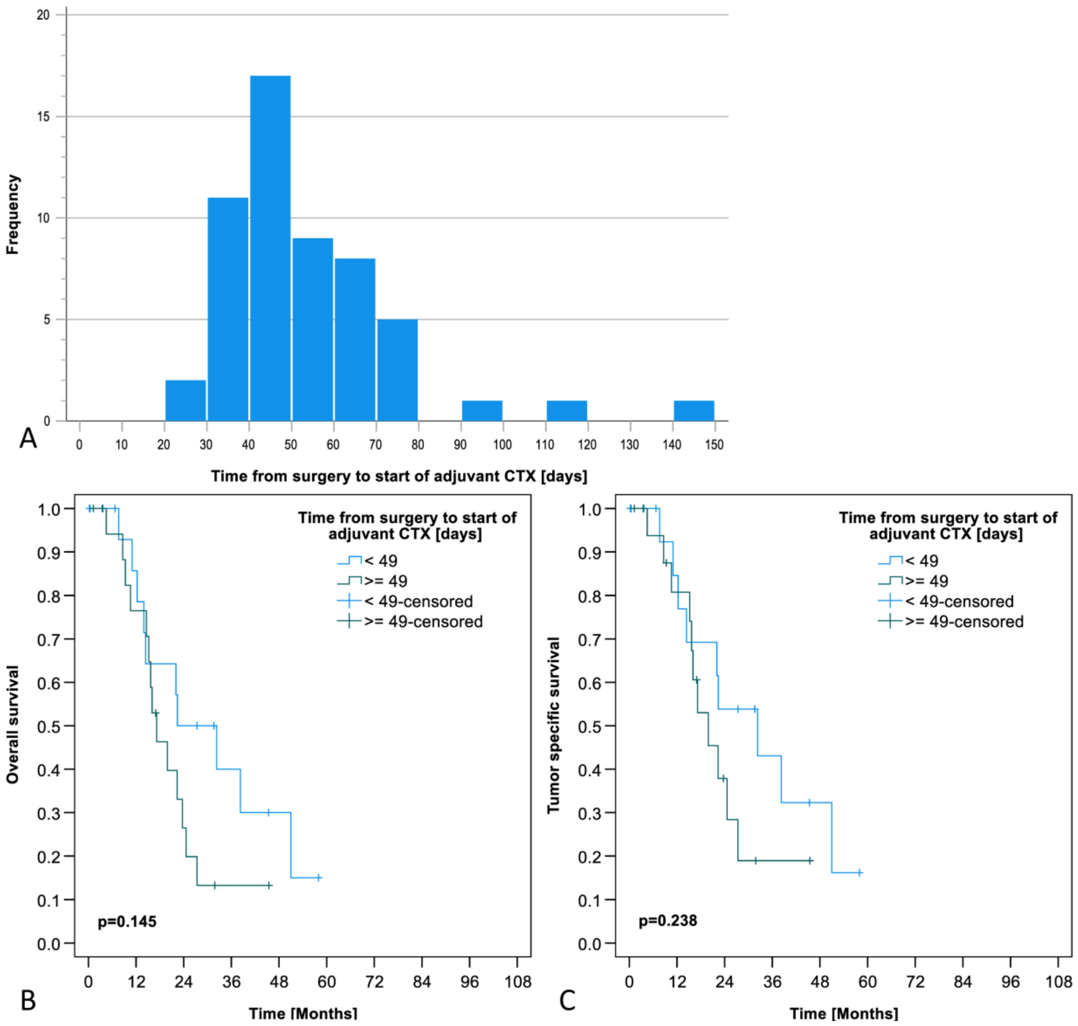

Post-hoc power calculation

A post-hoc power calculation was performed on the survival difference (overall survival) found for having received no versus any adjuvant chemotherapy within the TRG 3 subgroup. There were 44 cases included in the Cox regression with 33 events (deaths), resulting in an event rate of 0.75. These parameters were applied to the pwr_coxph procedure of the R package survivalpwr (8). The calculated power was 81.2%.

References

1. Lauren P. The Two Histological Main Types of Gastric Carcinoma: Diffuse and So-Called Intestinal-Type Carcinoma. An Attempt at a Histo-Clinical Classification. Acta Pathol Microbiol Scand. 1965;64:31-49.
2. Sobin LH, Gospodarowicz M, Wittekind C. TNM Classification of Malignant Tumours. 7 ed: Wiley-Blackwell.; 2009. pp.
3. Becker K, Mueller JD, Schulmacher C, Ott K, Fink U, Busch R, et al. Histomorphology and grading of regression in gastric carcinoma treated with neoadjuvant chemotherapy. Cancer. 2003;98(7):1521-30.
4. Warneke VS, Behrens HM, Boger C, Becker T, Lordick F, Ebert MP, et al. Her2/neu testing in gastric cancer: evaluating the risk of sampling errors. Ann Oncol. 2013;24(3):725-33.
5. Shitara K, Lordick F, Bang YJ, Enzinger P, Ilson D, Shah MA, et al. Zolbetuximab plus mFOLFOX6 in patients with CLDN18.2-positive, HER2-negative, untreated, locally advanced unresectable or metastatic gastric or gastro-oesophageal junction adenocarcinoma (SPOTLIGHT): a multicentre, randomised, double-blind, phase 3 trial. Lancet. 2023
6. Mathiak M, Warneke VS, Behrens HM, Haag J, Böger C, Krüger S, et al. Clinicopathologic Characteristics of Microsatellite Instable Gastric Carcinomas Revisited: Urgent Need for Standardization. Appl Immunohistochem Mol Morphol. 2017;25(1):12-24.
7. Benjamini Y, Hochberg Y. Controlling the False Discovery Rate: A Practical and Powerful Approach to Multiple Testing. Journal of the Royal Statistical Society: Series B (Methodological). 1995;57(1):289-300.
8. McGowan LDA. survivalpwr R package. p. https://lucymcgowan.github.io/survivalpwr/.
